# Digital and tomographic identification of risk indicators for gingival recession in the mandibular anterior region: A cross‐sectional study

**DOI:** 10.1002/jper.70073

**Published:** 2026-02-02

**Authors:** Gonzalo Blasi, Lise Maury, Lory Abrahamian, Youssra Abarchan, Behnam Taghavi, Jose Nart

**Affiliations:** ^1^ Department of Periodontology School of Dentistry Universitat Internacional de Catalunya Barcelona Spain; ^2^ Division of Periodontics Department of Advanced Oral Sciences and Therapeutics Baltimore School of Dentistry University of Maryland Baltimore Maryland USA; ^3^ Digital Dentistry Department Escuela Superior de Implantología y Rehabilitación Oral (ESIRO) Barcelona Spain

**Keywords:** cross‐sectional study, gingival recession, mandible, risk indicators

## Abstract

**Background:**

To assess clinical and anatomical risk indicators for mid‐buccal gingival recessions (GRs) in the mandibular anterior region using digital and tomographic tools.

**Methods:**

A cross‐sectional study was conducted on 103 patients (618 teeth). Clinical examination, intraoral scanning, and cone‐beam computed tomography (CBCT) were combined to record the keratinized tissue width (KTW), gingival phenotype, GR type (RT), probing pocket depth, clinical attachment level, recession depth (RD), tooth‐ridge angulation, tooth malpositioning (TM), buccal bone thickness (BBT), buccal bone dehiscence (BBD), root prominence (RP), gingival thickness (GT), and papilla height (PH). Associations with GR were analyzed using multilevel regression with generalized estimation equations.

**Results:**

Gingival recession was observed in 70.9% of patients and 38.3% of teeth, most often RT2. Central incisors were most affected. Univariate analysis associated GR to age, tooth type, TM, reduced KTW, decreased BBT, increased BBD, and RT, while thicker phenotypes showed protective effects. Multivariable analysis confirmed five independent predictors: age, tooth type, TM (OR = 3.11), reduced KTW (OR = 0.64), and greater BBD (OR = 1.64). RD increased with age (+0.03 mm/year) and BBD (+0.21 mm).

**Conclusions:**

Tooth malposition, inadequate KTW, and BBD are key risk indicators for gingival recession in the mandibular anterior region. Their identification is essential for preventive and therapeutic strategies.

**Plain language summary:**

Gingival recession, the apical migration of the gingival margin, is a common condition with implications for dental sensitivity, esthetics, and long‐term periodontal health. The lower anterior region is particularly susceptible, yet the anatomical and clinical factors contributing to this vulnerability are not fully defined using digital tools. In this cross‐sectional study of 103 patients (618 teeth), we integrated clinical examination, digital intraoral scanning, and CBCT to quantify soft and hard tissue characteristics. Recession was present in 70.9% of patients and 38.3% of teeth, predominantly affecting the central incisors. Multivariable analysis identified five independent risk indicators: older age, tooth type, tooth malposition, reduced keratinized tissue width, and buccal bone dehiscence. Recession depth increased with age and dehiscence severity. These findings underscore the critical role of both soft and hard tissue anatomy in determining susceptibility to gingival recession, providing a framework for risk assessment, preventive strategies, and individualized treatment planning in clinical practice.

## 1 INTRODUCTION

Gingival recession (GR) is defined as the apical displacement of the gingival margin from the cemento‐enamel junction (CEJ), resulting in the exposure of the root surface to the oral cavity.^1^ Although GR is not directly associated with tooth loss, it remains a significant clinical issue. It frequently presents as an esthetic concern and can lead to dentin hypersensitivity,^2^ radicular caries, non‐carious cervical lesions,^3^ and reduced oral health‐related quality of life.^4^

To better understand and manage this condition, gingival recession is classified into three recession types (RT1, RT2, and RT3) based on interproximal clinical attachment loss.^5^ This system distinguishes RT1 recessions, which lack interproximal attachment loss and are typically found in periodontally healthy patients, from RT2 and RT3 recessions.^6^, ^7^ In contrast, these latter types are characterized by concomitant interproximal attachment loss and are more prevalent in populations with poor oral hygiene and periodontal disease.^8^, ^9^

Indeed, the pathogenesis of gingival recession involves a localized inflammatory process influenced by a combination of risk indicators, which are broadly categorized as predisposing or precipitating factors.^10^ Predisposing factors are primarily anatomical or physiological conditions that create vulnerability, such as alveolar bone dehiscences.^11^ A thin gingival phenotype, in particular, significantly increases the risk of developing GR.^12^, ^13^ These inherent vulnerabilities are often triggered by precipitating factors, which include acquired habits or pathological conditions that inflict trauma on the gingiva. Common examples range from plaque‐induced inflammation to mechanical trauma from poorly designed dentures, improper toothbrushing techniques, or direct physical injury from oral piercings.^9^, ^14^, ^15^, ^16^

Among all anatomical regions, the mandibular anterior sextant is particularly susceptible to gingival recession due to the convergence of multiple risk‐enhancing features^8^, ^9^, ^17^: a thin buccal bone plate,^18^, ^19^ high frenulum attachments, and reduced vestibular depth.^1^ These factors coincide with high esthetic demands and frequent orthodontic intervention in this area, increasing its clinical relevance.^18^ Beyond its clinical importance, restricting the analysis to this sextant enhances methodological rigor by minimizing anatomical variability and functional heterogeneity across regions.^20^ Epidemiologic studies have reported GR in up to 90% of adult populations, with the mandibular anterior sextant frequently exhibiting the highest prevalence.^7^, ^9^, ^17^ In fact, in an Italian cohort, mandibular incisors were among the teeth with the highest prevalence of gingival recession ≥ 1 mm (17.49%) and among those showing the greatest proportions of RT2 and RT3 recessions.^9^

Despite the well‐documented susceptibility of the mandibular anterior region, there remains a lack of observational studies that specifically investigate risk indicators for gingival recession using integrated digital tools. In fact, previous studies have relied on conventional clinical assessments or two‐dimensional radiographic measurements, which are inherently limited by variability and reduced precision. By contrast, the integration of intraoral scanning (STL) with cone‐beam computed tomography (CBCT) has been validated as an accurate and reproducible method for three‐dimensional evaluation of soft and hard tissues, with mean registration errors below 0.2 mm.^21^, ^22^ In the field of periodontology, this integration has enabled non‐invasive quantitative analysis of periodontal and peri‐implant phenotypes.^23^, ^24^, ^25^ This approach allows reproducible morphometric quantification beyond the limits of conventional two‐dimensional assessments. Understanding these parameters is crucial, as it allows for early detection, effective prevention, and individualized treatment planning in this high‐risk area. Building on this rationale, the aim of the present cross‐sectional study was to identify risk indicators associated with mid‐buccal gingival recessions (GRs) in the mandibular anterior region through the integrated use of clinical examination, optical scanning, and CBCT imaging.

## 2 MATERIALS AND METHODS

### 2.1 Study design

This study was designed as a cross‐sectional observational study and reported in accordance with the STROBE guidelines.^26^

### 2.2 Study population and selection criteria

The study population consisted of patients recruited between November 2024 and May 2025 from the Department of Periodontology at the Universitat Internacional de Catalunya (UIC). Participants were those who required a mandibular CBCT scan as part of their existing treatment plan. To ensure a cross‐sectional assessment, the clinical examination and digital intraoral scan were performed at the same appointment or in close proximity to the CBCT acquisition. The study protocol was approved by the UIC Ethics Committee (PER ECL‐2024‐11) and conducted in accordance with the Declaration of Helsinki (1975, revised in 2024).

Eligible participants were systemically healthy, ≥ 18 years, non‐smokers, periodontally healthy/stable according to the AAP/EFP definition,^27^, ^28^ with six natural teeth in the mandibular anterior region, and a large‐field mandibular CBCT scan.

On the other hand, subjects were excluded in the following cases: systemic conditions or medication that could interfere with the periodontium, pregnancy, poor quality CBCT, prior soft tissue augmentation in the area, ongoing orthodontic therapy, or failure to provide consent. Prior to enrollment, all patients received detailed information and provided written informed consent.

### 2.3 Clinical outcomes

As part of the clinical evaluation, the following parameters were recorded for each tooth:

1. Gingival phenotype, assessed as thin‐scalloped, thick‐scalloped, or thick‐flat.^29^ The determination of thickness (“thin” vs. “thick”) was based on the transparency of the periodontal probe (PCP UNC 15, Hu‐Friedy, Chicago, IL, USA) when inserted into the mid‐buccal sulcus. If the probe outline was visible through the tissue, it was categorized as “thin,” and if not, as “thick.”
2. Presence or absence of gingival recession, identified as the apical displacement of the gingival margin in relation to the CEJ.^1^
3. Classification of the type of GR defect into RT1, RT2, or RT3.^5^
4. Mid‐buccal probing pocket depth (PPD) measured with a periodontal probe (PCP UNC 15, Hu‐Friedy, Chicago, IL, USA) and rounded up to the nearest 0.5 mm.
5. Mid‐buccal clinical attachment level (CAL) obtained as RD + PPD.

### 2.4 Digital intra‐oral scanning outcomes

**FIGURE 1:**
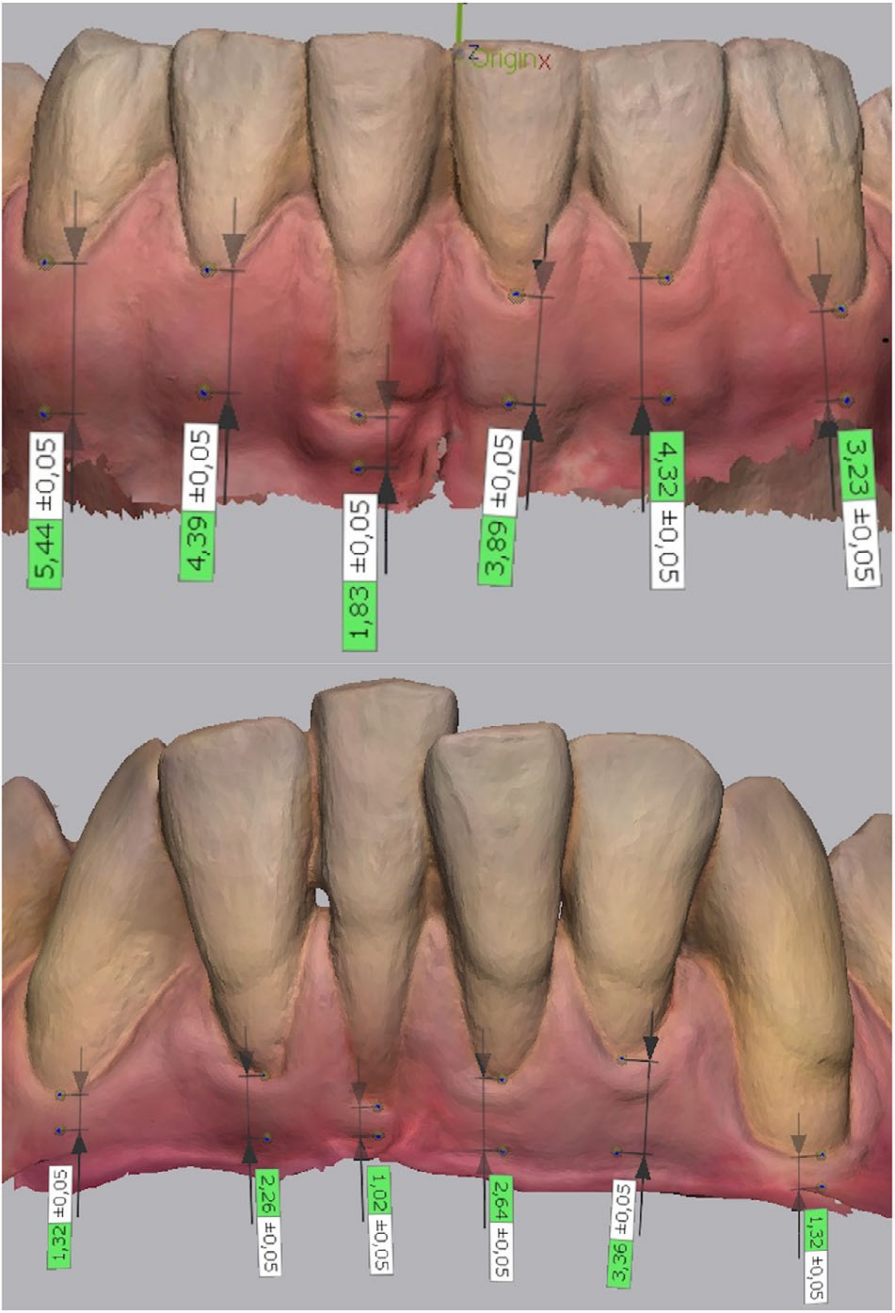
Digital measurements of keratinized tissue width using Geomagic (Control X). Linear measurements were performed on the digital models to quantify keratinized tissue.

Mandibular arches were scanned using an intraoral scanner (3Shape Trios, Copenhagen, Denmark) to generate standard tessellation language (STL) and polygon (PLY) files. These files were anonymized and imported into Geomagic Control X (Geomagic, 3D Systems, Research Triangle Park, NC, USA). A pre‐calibrated examiner (Lory Abrahamian) recorded the mid‐buccal recession depth (RD), keratinized tissue width (KTW) (Figure 1), tooth rotation (TR); defined as a noticeable mesiolingual or distolingual displacement of the tooth around its horizontal axis, and tooth malposition (TM); characterized as buccal positioning of the tooth with respect to the adjacent teeth. To prevent detection bias, this digital examiner (Lory Abrahamian) was blinded to the clinical and CBCT findings, which were gathered by separate operators.

### 2.5 CBCT outcomes

**FIGURE 2:**
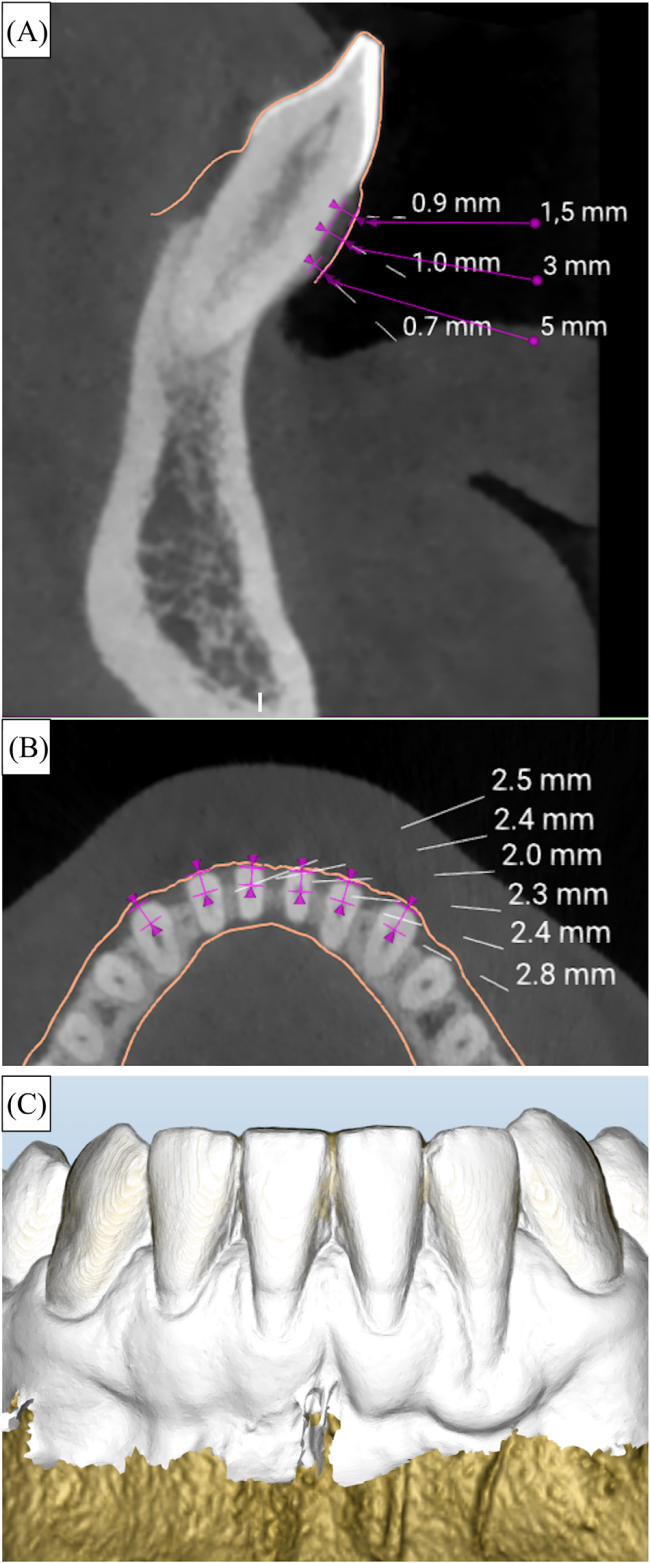
Radiographic measurements using cone‐beam computed tomography (CBCT). (A) Gingival thickness measured at 1.5, 3, and 5 mm from the gingival margin. (B) Root prominence assessment. (C) Superimposition of standard tessellation language (STL) and Digital Imaging and Communications in Medicine (DICOM) files for integrated digital analysis.

All CBCT examinations had been previously acquired for diagnostic or treatment planning purposes (implantology, orthodontics, or endodontics) in accordance with the ALARA principle.^30^ No additional radiographic exposures were performed for this study. CBCT scans were carried out by a dental radiologist using a cone‐beam 3D imaging system (I‐CAT Platinum CT unit, Imaging Sciences International, Hatfield, PA, USA) with the following exposure settings: kVp = 120, mA = 5, mAs = 18.54, 8.9 s, and 0.3 voxels. The CBCT scans were exported in Digital Imaging and Communications in Medicine (DICOM) files. A single pre‐calibrated operator (Youssra Abarchan) analyzed the CBCT data using an implant planning program (RealGUIDE Software Suite, ZimVie, Palm Beach Gardens, FL, USA) to evaluate the following radiographic outcomes of interest (Figure 2).

1. Buccal bone thickness (BBT), was measured on axial CBCT scans at 0.5 mm apical to the crest of the buccal bone;
2. Buccal bone dehiscence (BBD), was calculated as the vertical distance from the CEJ to the buccal bone crest in the midfacial view;
3. Tooth‐ridge angulation (T‐RA), was measured by tracing a line along the long axis of the tooth—from the incisal edge to the root apex—and a second line from the gnathion, selected as a fixed anatomic landmark on the chin. The angle between these two lines was then calculated.
4. Root prominence (RP), was determined by tracing a line from the buccal bony contour to the mid‐buccal part of the root at 2 mm from the CEJ on the axial scan.

Additionally, the STL files were imported to the same planning software and superimposed with the DICOM files. The following parameters were assessed:

1. Gingival thickness (GT) was measured in the midfacial CBCT view at 1.5, 3, and 5 mm from the gingival margin.
2. Mesial and distal papilla height (MPH and DPH, respectively), assessed as the vertical distance between the tip of the interproximal papilla and the interproximal bone crest.

### 2.6 Sample size calculation

The sample size was calculated to detect significant associations between anatomical predictors and gingival recession using logistic regression. The KTW was chosen as the reference predictor, as Mascardo et al.^13^ identified it as the most significant predictor in their logistic model. They reported an odds ratio (OR) of 0.62 for KTW, with a mean of 4.47 mm and SD of 1.59 mm, which allowed calculation of a clinically relevant change. Using Demidenko's approach for logistic regression^31^ in GPower (GPower software, Düsseldorf, Germany), 135 independent teeth were required for 80% power at a 95% confidence level. To account for clustering of teeth within patients, the design effect was calculated as D = (m‐1) × ICC +1. With m = 6 teeth per patient and an ICC of 0.5, the resulting design effect was 3.5. This resulted in a final requirement of 473 dependent teeth, corresponding to approximately 103 patients, ensuring adequate power while accounting for the clustered data structure.

### 2.7 Statistical analysis

Descriptive statistics were reported as absolute and relative frequencies for categorical variables and mean ± SD for continuous variables. Associations between GR and independent variables were initially assessed using binary logistic regression, with odds ratios (ORs) and 95% confidence intervals obtained via the Wald Chi‐square test. Variables with *p* < 0.05, as well as clinically relevant variables with *p* < 0.1, were entered into multivariable models.

To account for clustering of teeth within patients, generalized estimating equations (GEEs) were used. Given the large and balanced dataset, in which most patients contributed six mandibular anterior teeth, models were fitted using a robust Huber–White sandwich‐type estimator under an independence working correlation structure.^32^, ^33^, ^34^ Sensitivity analyses using exchangeable and unstructured correlation matrices yielded comparable estimates. Additionally, the Quasi‐likelihood under the Independence Model Criterion (QIC) did not indicate improvement, supporting the robustness of the independence‐based specification.

Variable selection followed a two‐step procedure: simple GEE models were first fitted for each predictor, and variables with *p* < 0.05 or *p* < 0.1 if clinically relevant were included in the multivariable GEE model. This combined statistical–clinical approach ensured retention of important predictors while avoiding overfitting. Given the anatomical relationships among several predictors, potential multicollinearity was evaluated through pairwise correlation analysis. Analyses were performed using SPSS version 29.0.1.1, with a significance level of 5%.

## 3 RESULTS

### 3.1 Participants and teeth characteristics

The present study analyzed a cohort of 103 patients (mean age 49.1 ± 15.2 years, 67 females and 35 males) comprising a total of 618 teeth. Mandibular anterior GRs were identified in 70.9% at patient level (95% CI: 62.1%–79.7%) and 38.3% at tooth level (95% CI: 34.5%–42.2%). Regarding the classification of GR, 43.0% were categorized as recession type RT1, 53.2% RT2, and 3.8% RT3.

The mean PPD was 2.17 ± 0.71 mm, while the mean KTW was 3.83 ± 1.47 mm. The average BBT was 0.56 ± 0.42 mm, and the mean BBD measured 3.93 ± 2.07 mm. The RP averaged 0.62 ± 0.79 mm. Patient, clinical, digital, and radiographic measures are summarized in Table 1.

**TABLE 1:** Description of patient, clinical, digital, and radiographic site variables for the 103 patients and 618 included teeth.

Patient‐level variables	Mean (*n* = 103)
**Sex**	
Male	36 (35.0%)
Female	67 (65.0%)
**Age** (years)	49.1 ± 15.2

Clinical‐level variables	Mean (*n* = 618)
**Gingival phenotype**	
Thin scalloped	24 (23.3%)
Thick scalloped	65 (63.1%)
Thick flat	14 (13.6%)
**Teeth with GR** (%)	38.3 ± 35.3
**Recession type**	
RT1	102 (43.0%)
RT2	126 (53.2%)
RT3	9 (3.8%)
**PPD** (mm)	2.17 ± 0.71
**CAL** (mm)	2.83 ± 1.24

Digital‐level variables	Mean (*n* = 618)
**RD** (mm)	0.67 ± 1.10
**Tooth rotation** (yes)	209 (34.0%)
**Tooth malposition** (yes)	161 (26.2%)
**KTW** (mm)	3.83 ± 1.47

CBCT‐level variables	Mean + SD (*n* = 618)
**BBT** (mm)	0.56 ± 0.42
**BBD** (mm)	3.93 ± 2.07
**Root prominence** (mm)	0.62 ± 0.79
**Tooth–ridge angulation** (°)	17.93 ± 6.28
**GT 1.5 mm** (mm)	1.11 ± 0.31
**GT 3 mm** (mm)	1.07 ± 0.42
**GT 5 mm** (mm)	0.82 ± 0.37
**Mesial papilla height** (mm)	3.38 ± 0.64
**Distal papilla height** (mm)	3.62 ± 0.73

Abbreviations: BBD, buccal bone dehiscence; BBT, buccal bone thickness; CAL, clinical attachment level; CBCT, cone‐beam computed tomography; GR, gingival recession; GT, gingival thickness; KTW, keratinized tissue width; PPD probing pocket depth; RD, recession depth; RT, recession type.

### 3.2 Risk indicators for mid‐buccal GR

#### 3.2.1 Univariate logistic regression analysis of factors associated with GR

The analysis showed that age had a significant effect on the presence of gingival recession (OR = 1.04; 95% CI: 1.02–1.06; *p* < 0.001). Each additional year of age increased the odds of GR by approximately 4%, with mean ages of 54.2 years in teeth with GR compared with 45.9 years in those without recession. Gingival phenotype also influenced risk (*p* = 0.031), with thick scalloped and thick flat phenotypes reducing the odds of GR by 57% and 67%, respectively, relative to the thin phenotype. In terms of prevalence, GR was observed in 54.9% of thin, 34.4% of thick scalloped, and 28.6% of thick flat cases.

Tooth type emerged as another important determinant (*p* < 0.001). Lateral incisors and canines showed lower odds of gingival recession compared with central incisors (OR = 0.53; 95% CI: 0.38–0.75 and OR = 0.34; 95% CI: 0.22–0.511, respectively). Furthermore, lateral incisors presented a significantly higher risk of GR compared with canines (OR = 1.57; *p* = 0.004), consistent with prevalence rates of 51.9%, 36.4%, and 26.7% across the three tooth types. PPD showed a slight protective effect (OR = 0.70; 95% CI: 0.49–1.00; *p* = 0.050), as the mean PPD was slightly lower in teeth with GR (2.06 mm) compared with those without (2.24 mm). As expected, CAL was strongly associated with the presence of GR (OR = 8.38; 95% CI: 5.58–12.6; *p* < 0.001).

Digital variables also showed significant associations. Malpositioning increased the odds of GR nearly six‐fold (OR = 5.78; 95% CI: 3.44–9.72; *p* < 0.001), with prevalence rates of 68.9% in malpositioned teeth compared with 27.8% in properly aligned teeth. The KTW demonstrated a significant protective effect (OR = 0.60; 95% CI: 0.49–0.75; *p* < 0.001), with each additional millimeter reducing the odds of GR by 40%. Similarly, BBT showed a strong protective effect (OR = 0.16; 95% CI: 0.06–0.46; *p* = 0.001), corresponding to an 84% reduction in risk per additional millimeter. By contrast, BBD was associated with higher odds of GR (OR = 1.77; 95% CI: 1.44–2.19; *p* < 0.001), as was RP (OR = 1.70; 95% CI: 1.30–2.21; *p* < 0.001).

Regarding GT, only the measurement at 1.5 mm reached statistical significance (OR = 0.24; 95% CI: 0.08–0.78; *p* = 0.018), indicating a 76% reduction in odds of GR per additional millimeter. For PH, only mesial measurements suggested a potential influence (OR = 1.45; 95% CI: 0.99–2.11; *p* = 0.055), with higher values recorded in teeth presenting GR (Table 2).

**TABLE 2:** Association of patient, clinical, radiographic, and digital variables with the presence of gingival recession (GR) using univariable logistic regression (GEE).

Variable	OR	95% CI	*p*‐value
**Age (years)**	1.04	1.02–1.06	**<0.001** ^***^
**Sex**			
Female	1.16	0.62–2.15	0.644
Male	1		
**Tooth type**			**<0.001** ^***^
Central incisor	1		
Lateral incisor	0.53	0.38–0.75	**<0.001** ^***^
Canine	0.34	0.22–0.51	**<0.001** ^***^
**Gingival phenotype**			0.031^*^
Thin scalloped	1		
Thick scalloped	0.43	0.22–0.86	**0.017** ^*^
Thick flat	0.33	0.12–0.89	**0.029** ^*^
**Malposition** (yes)	5.78	3.44–9.72	**<0.001** ^***^
**PPD** (mm)	0.70	0.49–1.00	0.050
**CAL** (mm)	8.38	5.58–12.6	**<0.001** ^***^
**KTW** (mm)	0.60	0.49–0.75	**<0.001** ^***^
**BBD** (mm)	1.77	1.44–2.19	**<0.001** ^***^
**BBT** (mm)	0.16	0.06–0.46	**0.001** ^**^
**Root prominence** (mm)	1.70	1.30–2.21	**<0.001** ^***^
**GT 1.5 mm** (mm)	0.24	0.08–0.78	**0.018** ^*^
**GT 3 mm** (mm)	0.56	0.26–1.19	0.133
**GT 5 mm** (mm)	1.59	0.80–3.17	0.188
**Mesial papilla height** (mm)	1.45	0.99–2.11	0.055
**Distal papilla height** (mm)	1.13	0.83–1.55	0.442

Abbreviations: BBD, buccal bone dehiscence; BBT, buccal bone thickness; CAL, clinical attachment level; GT, gingival thickness; KTW, keratinized tissue width; PPD, probing pocket depth.

* *p* < 0.05.

** *p* < 0.01.

*** *p* < 0.001.

#### 3.2.2 Multivariable logistic regression analysis of factors associated with GR

In the multivariable logistic regression model adjusted for confounding variables, five independent predictors remained significantly associated with the presence of gingival recession. Age showed a positive association, with each additional year increasing the odds of recession by 6% (OR = 1.06; 95% CI: 1.03–1.08; *p* < 0.001). Tooth type remained influential, with lateral incisors and canines showing lower odds of recession than central incisors (OR = 0.55 and OR = 0.15, respectively; both *p* < 0.01). TM more than tripled the odds of recession (OR = 3.11; 95% CI: 1.65–5.87; *p* < 0.001).

Finally, the KTW demonstrated a significant protective effect, with each millimeter increase reducing the odds of recession by 36% (OR = 0.64; 95% CI: 0.48–0.85; *p* = 0.002), whereas greater BBD increased the odds (OR = 1.64; 95% CI: 1.32–2.05; *p* < 0.001).

After adjustment, the protective effect of GT was no longer statistically significant. No statistically significant interaction was found between BBD and RP (*p* = 0.549), or between RP and T‐RA (*p* = 0.166).

As part of the model evaluation, pairwise correlations among anatomical variables indicated only moderate associations between BBT and BBD/GT. A reduced auxiliary model excluding BBT and GT yielded estimates comparable to the main model, confirming that multicollinearity did not materially influence the stability or interpretation of the regression results. All results are presented in Table 3.

**TABLE 3:** Risk indicators of gingival recession (GR) from the multivariable logistic regression model with generalized estimating equation (GEE).

Variable	OR	95% CI	*p*‐value
**Age (years)**	1.06	1.03–1.08	<0.001^***^
**Tooth type**			
Central incisor	1		
Lateral incisor	0.55	0.36–0.86	**0.009** ^**^
Canine	0.15	0.07–0.31	**<0.001** ^***^
**Gingival phenotype**			
Thin scalloped	1		
Thick scalloped	0.88	0.33–2.33	0.793
Thick flat	0.77	0.20–2.97	0.703
**Malposition** (yes)	3.11	1.65–5.87	**<0.001** ^***^
**PPD** (mm)	0.94	0.62–1.40	0.746
**KTW** (mm)	0.64	0.48–0.85	**0.002** ^**^
**BBD** (mm)	1.64	1.32–2.05	**<0.001** ^***^
**BBT** (mm)	1.72	0.52–5.59	0.375
**Root prominence** (mm)	1.51	0.95–2.41	0.084
**GT 1.5 mm** (mm)	0.72	0.17–3.14	0.666
**Mesial papilla height** (mm)	1.44	0.81–2.54	0.216

Abbreviations: BBD, buccal bone dehiscence; BBT, buccal bone thickness; GT, gingival thickness; KTW, keratinized tissue width; PPD, probing pocket depth.

** *p* < 0.01.

*** *p* < 0.001.

### 3.3 Severity of GR

#### 3.3.1 Univariable predictors of recession severity

Several variables demonstrated significant associations with increasing GR depth. Age was positively associated with severity, with each additional year linked to an increase of 0.02 mm in RD (*p* = 0.038). The presence of BBD was a strong predictor of deeper recession (*β* = 0.27; 95% CI: 0.17–0.37; *p* < 0.001), and RP also showed a significant association (*β* = 0.19; 95% CI: 0.00–0.37; *p* = 0.047). Additionally, a greater T‐RA was associated with increased RD (*β* = 0.02; 95% CI: 0.00–0.47; *p* = 0.033), and GT5 was positively correlated with RD (*β* = 0.54; 95% CI: 0.09–0.98; *p* = 0.019) (Table 4).

**TABLE 4:** Univariable and multivariable linear regression for severity of gingival recession (GR).

	Univariable linear regression			Multivariable linear regression		
Variable	*β*	95% CI	*p‐v*alue	*β*	95% CI	*p‐*value
**Age** (years)	0.02	0.00–0.03	**0.038** ^*^	0.03	0.01–0.04	**<0.001** ^***^
**BBD** (mm)	0.27	0.17–0.37	**<0.001** ^***^	0.21	0.10–0.32	**<0.001** ^***^
**BBT** (mm)	−0.33	−0.67 – 0.02	0.065	—	—	—
**Malposition** (yes)	0.32	−0.04 – 0.67	0.078	0.09	−0.20 – 0.37	0.542
**Tooth–ridge angulation** (°)	0.02	0.00–0.47	**0.033** ^*^	0.01	−0.1 – 0.04	0.228
**Root prominence** (mm)	0.19	0.00–0.37	0.047^*^	0.06	−0.13 – 0.25	0.531
**PPD** (mm)	−0.05	−0.32 – 0.23	0.744	—	—	—
**CAL** (mm)	0.72	0.66–0.79	<0.001^***^	—	—	—
**KTW** (mm)	−0.16	−0.33 – 0.01	0.072	—	—	—
**GT 5 mm** (mm)	0.54	0.09–0.98	0.019^*^	0.25	−0.2 – 0.69	0.276

Abbreviations: BBD, buccal bone dehiscence; BBT, buccal bone thickness; CAL, clinical attachment level; GT, gingival thickness; KTW, keratinized tissue width; PPD, probing pocket depth.

* *p* < 0.05.

*** *p* < 0.001.

#### 3.3.2 Multivariable model of recession severity

In the adjusted multivariable linear regression model, only age and BBD remained significantly associated with the depth of gingival recession. Age demonstrated a consistent relationship, with each year corresponding to a 0.03 mm increase in RD (*p* < 0.001). BBD retained a strong positive association (*β* = 0.21; 95% CI: 0.10–0.32; *p* < 0.001). Other variables (including RP, T‐RA, and GT5) did not remain significant in the adjusted model (Table 4).

## 4 DISCUSSION

The primary contribution of this investigation is the use of an integrated digital and tomographic workflow to provide precise, three‐dimensional quantification of risk indicators for gingival recession. Our findings confirm a substantial patient‐level prevalence of GR at 70.9%. This figure is consistent with the high prevalence rates reported in U.S. (91.6%), European (57.2%), and South American adult (> 90%) cohorts, although our study specifically focused on this high‐risk anatomical sextant.^7^, ^9^, ^17^ However, interpretation of recession prevalence across epidemiologic studies should be made cautiously, as variability often stems not only from differences in diagnostic criteria but also from methodological factors and population characteristics.

The main finding was that age, tooth type, TM, a reduced KTW, and the presence of a BBD were significant risk indicators for the presence of gingival recession. Furthermore, the severity of the recession was significantly associated with increasing age and BBD. These observations align with and extend previous epidemiological studies by providing quantitative estimates derived from three‐dimensional measurements.^17^, ^35^ Consistent with the broader literature, age proved to be a significant factor, with each additional year increasing the odds of GR presence by 6% (OR = 1.06) and the RD by 0.03 mm. This finding likely reflects the cumulative, long‐term effect of precipitating factors such as mechanical trauma from toothbrushing and chronic, low‐grade plaque‐induced inflammation.^8^, ^15^ The study also reinforced the concept that local anatomy is a key predisposing factor. Specifically, central incisors were at the highest risk compared with lateral incisors and canines, which corroborates large‐scale epidemiological studies that identified both incisors and the mandibular arch as primary risk indicators for RT1 GR.^17^, ^35^

A central finding of this study was that tooth malposition emerged as a potent risk indicator, more than tripling the odds of presenting a recession (OR = 3.11). This observation strongly supports the established biological rationale that positioning a tooth outside its natural alveolar housing leads to the formation of a bone dehiscence, creating a “*locus minoris resistentiae*” for the development of gingival recession.^19^ The clinical relevance of this mechanism is most intensely debated in the context of orthodontic therapy. Several studies suggest a strong link; for instance, Renkema et al. found that orthodontic patients had over four times the odds of developing recessions compared with untreated controls,^36^ and Celis et al. noted that recession incidence increased dramatically during the long‐term retention phase.^37^ However, the relationship is not absolute. Foundational animal studies by Wennström et al. demonstrated that labial tooth movement could induce recession, but significant attachment loss occurred only when inflammation was also present, underscoring its role as a key co‐factor.^38^ Human data similarly indicate that even substantial incisor advancement does not inherently increase recession risk when orthodontic movement occurs within a healthy periodontal environment.^39^, ^40^

Our findings help to clarify this by underscoring the critical interplay between TM, RP, and the resulting BBD. The data suggest that the crucial anatomical factor is not the overall size of the jaw, but rather the specific position of the tooth root within its immediate bony housing.^18^ When a tooth is malpositioned buccally, especially one with a prominent root convexity, it is physically displaced to the limit of, or beyond, the alveolar bone envelope. This creates the BBD, which our model identified as one of the most robust predictors for both the presence (OR = 1.64) and severity (*β* = 0.21) of gingival recession. This cascade, where malposition and prominence lead to a dehiscence, establishes the ultimate anatomical vulnerability. Regardless of the initial cause, the dehiscence represents the final common pathway, leaving the overlying soft tissue without vital osseous support and susceptible to breakdown from subsequent inflammatory or mechanical insults.^13^ This is supported by animal studies, such as Danz et al., which showed that facial tooth movement in rats consistently led to the development of BBD, even though the development of actual gingival recession was scarce.^41^ The fact that BBD was one of the most robust predictors for both the presence (OR = 1.64) and severity (*β* = 0.21) of gingival recession in our model underscores that this absence of underlying buccal bone is the ultimate anatomical defect that predisposes the gingival margin to apical migration.

The novel contribution of the present study lies not in the identification of keratinized tissue width as a risk indicator per se, but in the quantification of its protective effect through three‐dimensional, reproducible methods. Specifically, each additional millimeter of KTW reduced the odds of GR by 36% (OR = 0.64). The importance of this factor, particularly in orthodontics, was highlighted by Mandelli et al., who found that the baseline KTW was significantly correlated with the prevalence, extension, and severity of gingival recession after treatment.^42^ This is further supported by Celis et al., who also identified a thin phenotype as a key risk indicator for post‐orthodontic recessions.^37^ Regarding GT, although it is often anatomically correlated with KTW—where a wider zone of keratinized tissue frequently corresponds to a thicker phenotype^43^, ^45^—these parameters represent distinct dimensions. Interestingly, while GT was significant in the univariate analysis, it lost significance in the multivariable model. This lack of independent predictive value is likely not due to sample size, but rather because its effect is mediated by the underlying BBD. The presence of a BBD represents the lack of osseous support, which is the primary anatomical determinant of recession. Once BBD is included in the model, it acts as the dominant variable, overriding the secondary protective effect of soft tissue thickness. Thus, BBD and KTW emerge as the most robust independent predictors.

The present findings corroborate previously reported associations between morphometric variables and gingival recession, while reinforcing the value of integrating intraoral scanning and CBCT imaging for quantitative assessment of soft‐ and hard‐tissue dimensions. Early identification of these factors through digital workflows may improve patient counseling, risk stratification, and preventive strategies, especially in orthodontic cases where periodontal vulnerability must be carefully considered.

The strengths of this study lie in its methodological approach, integrating intraoral scanning with CBCT to generate precise, reproducible three‐dimensional datasets. This workflow provided objective digital validation of risk indicators in a high‐risk anatomical region, minimized examiner‐dependent variability, and enabled the simultaneous evaluation of both soft‐ and hard‐tissue parameters.^44^, ^45^ The accuracy of this digital approach has been further supported by Lee et al., who reported that digital scanning provides greater precision than traditional periodontal probing in the assessment of keratinized tissue width.^46^ Nevertheless, some limitations should be acknowledged. The primary constraint is the cross‐sectional nature of the study design, which identifies strong associations but does not allow for establishing a cause‐and‐effect relationship. Second, key factors influencing gingival recession, such as oral hygiene measures, history of orthodontic treatment, and the presence of restorations, were not systematically assessed. Future studies should include these variables to better account for confounders. Third, while the exclusive focus on the mandibular anterior region is clinically justified by its high susceptibility to gingival recession, this specificity may limit generalizability to other regions of the dentition. Fourth, interproximal bone crest levels were not directly measured; instead, papilla height relative to the crest was used as a proxy, which may limit the interpretation of interproximal support. Future studies should incorporate the alveolar bone crest position to better clarify this relationship. Additionally, limitations inherent to CBCT resolution in detecting very thin bone morphotypes should be acknowledged, as should the single‐center, university‐based nature of the cohort. Finally, including only patients with pre‐existing CBCTs may have introduced selection bias toward individuals undergoing orthodontic or implant‐related treatments, who could have higher treatment demands or specific anatomical characteristics and may therefore not be fully representative of the general population.

## 5 CONCLUSION

Within the limitations of this study, age, tooth type, tooth malposition, BBD, and reduced KTW were identified as significant risk indicators for mid‐buccal GR in the mandibular anterior region.

## AUTHOR CONTRIBUTIONS

**Gonzalo Blasi**: Contributed to the study conception; data interpretation; drafted and critically revised the manuscript. **Lise Maury** and **Lory Abrahamian**: Contributed equally to data collection; data interpretation; manuscript writing, and critical revision. **Youssra Abarchan** and **Behnam Taghavi**: Assisted with data collection. **Jose Nart**: Provided study supervision and contributed to manuscript revision. All authors have read and approved the final version of the manuscript.

## CONFLICT OF INTEREST STATEMENT

The authors declare no conflicts of interest related to the content of this manuscript.
